# Understanding the economic burden of chronic cough: a systematic literature review

**DOI:** 10.1186/s12890-023-02709-9

**Published:** 2023-10-31

**Authors:** Vishal Bali, Ada Adriano, Aidan Byrne, Katherine G. Akers, Andrew Frederickson, Jonathan Schelfhout

**Affiliations:** 1 Center for Observational and Real-World Evidence (CORE), Merck & Co., Inc, Rahway, NJ USA; 2 Outcomes research, MSD, London, UK; 3 PRECISIONheor, New York, NY USA; 4 Merck Sharp & Dohme Corp, 2000 Galloping Hill Road, Kenilworth, NJ 07033 US

**Keywords:** Chronic cough, Refractory chronic cough, Unexplained chronic cough, Burden, Economic burden

## Abstract

Chronic cough (CC) is associated with high healthcare resource utilization (HCRU) due to challenges in diagnosis and treatment and is anticipated to have a substantial economic impact. This systematic literature review (SLR) sought to identify evidence on the cost-effectiveness of treatments and the economic burden associated with CC. Electronic database searches were supplemented with searches of conference proceedings and health technology assessment body websites. Two independent reviewers assessed all citations for inclusion based on predefined inclusion/exclusion criteria. Key inclusion criteria were patient population with CC, and outcomes related to cost-effectiveness and HCRU and costs. After screening, one cost-effectiveness analysis was identified, alongside eight studies reporting HCRU and costs related to CC. Though evidence was limited, studies suggest that patients with CC incur higher costs and use more resources than those with acute cough. Types of resource use reported included healthcare contacts and prescriptions, diagnostic tests, referrals and specialist evaluations, and treatment use. There is a paucity of literature on HCRU and costs in CC, and very limited cost-effectiveness analyses. The economic burden appears higher in these patients however, without direct comparison to the general population it is difficult to determine the total impact. The increased burden is expected to be a result of the challenges with diagnosis and lack of approved treatments. However, limited conclusions can be drawn in the absence of further data. Future studies should endeavor to quantify the HCRU and cost attributable to patients with CC.

**Supplementary Information:**

The online version contains supplementary material available at 10.1186/s12890-023-02709-9.

## Take home message

Patients with CC encounter multiple physicians and specialists to seek a definitive diagnosis, resulting in delayed diagnosis and appropriate treatment. This SLR reported a paucity of economic data; however, it found patients with CC to incur higher costs and more resource use than those with acute cough.

**Supplementary Information:**

The online version contains supplementary material available at 10.1186/s12890-023-02709-9.

## Background

Chronic cough (CC) is typically defined as a cough persisting for more than eight weeks [1–4]. Although cough is a common reflex mechanism, excessive and prolonged cough can be highly burdensome to patients, disrupting daily activities for the individual and those around them [2, 4, 5]. In severe cases, patients with CC may experience complications such as stress urinary incontinence, interference with speech, depression, and syncope, which can have a substantial negative impact on quality of life (QoL) and on activities of daily living [4, 6].

Many patients with CC are believed to have cough reflex hypersensitivity, in which the reflex response occurs in response to low levels of stimulation from thermal, chemical or mechanical sources [4]. In some cases, CC is associated with an underlying comorbid condition, such as asthma, gastroesophageal reflux disease (GERD), or upper airway cough syndrome (UACS). When cough persists despite treatment of the associated condition, this is defined as refractory chronic cough (RCC) [6]. Conversely, unexplained chronic cough (UCC) may be diagnosed when all other aetiologies have been excluded through a thorough clinical evaluation and work-up [7]. In both RCC and UCC, cough reflex hypersensitivity has been proposed to contribute to the pathophysiology of CC [6, 8]. This review focuses on CC, inclusive of RCC and UCC.

CC has been estimated to affect around 10% of the adult population [5, 9]. A pooled analysis estimated an overall prevalence of 9.6%, with higher regional prevalence rates in Oceania (18.1%), Europe (12.7%), and America (11.0%), than in Asia (4.4%) and Africa (2.3%) [5]. Overall, population-based prevalence estimates of CC are limited [9].

Patients presenting with CC progress through evidence-based assessments to evaluate and diagnose treatable traits of the disease in attempts to offer direct therapy [4]. Patients often interact with multiple physicians and receive several specialist referrals in an attempt to seek a definitive diagnosis [10–12]. Until 2021, there was no CC specific diagnosis code [13]. Delays to diagnosis and appropriate treatment may therefore occur as different therapeutic options are tried [6]. The European Respiratory Society (ERS) guidelines recommend sequential therapeutic trials of a number of agents in turn, with treatment ceased if no responses are observed [4]. Currently trial therapies include antitussives, protussives, inhaled corticosteroids (ICS), bronchodilators, and neuromodulators, such as opioids, pregabalin, and gabapentin; however, none of these are currently approved for use in CC [4]. Indeed, at the time of writing, there are no approved pharmacological treatments for CC, however, a number of clinical trials investigating novel treatments for CC are underway [4].

Given the increased healthcare resource utilization (HCRU) required to determine a diagnosis of CC and trial of different therapeutic interventions, CC is expected to have a substantial economic impact. This may include both direct costs such as physician costs, medication costs, diagnosis costs and hospitalization, as well indirect costs resulting from productivity losses and absenteeism from work. There is currently limited evidence on the economic burden of CC, and in particular the burden specifically attributable to RCC and UCC. The lack of approved therapies for CC may contribute to the paucity of economic evaluations in this therapy area. A systematic literature review (SLR) was performed to identify evidence on the cost-effectiveness of treatments and the economic burden associated with respect to HCRU and costs attributable to CC.

## Methods

### Search strategy

A search was performed on 25 February 2021 in the MEDLINE, EMBASE, EconLit and EBM Reviews electronic databases via the Ovid platform. A combination of free text searching, and subject headings were used to capture the target population and outcomes. Study design filters for economic evidence recommended by the Scottish intercollegiate Guidelines Network (SIGN) were used, [14] supplemented with terms from alternative search strategies recommended by the InterTASC Information Specialists’ Sub-Group Search Filter Resource to increase the sensitivity of the search [15]. The complete search strings are presented in Supplementary Tables 1, Supplementary Tables 2, Supplementary Tables 3, and Supplementary Table 4.

The NHS Economic Evaluation Database, Health Economic Evaluations Database, and Tufts Cost-Effectiveness Analysis Registry were hand-searched using key population search terms to identify relevant studies. Hand searches of conference proceedings of annual meetings of relevant societies from the two years prior to search date were used to augment the database searches. Societies included the American Academy of Allergy, Asthma, and Immunology, American College of Allergy, Asthma, and Immunology, American College of Chest Physicians, American Thoracic Society, and European Respiratory Society. Health technology assessments (HTA) evaluating therapies for CC as published by the National Institute of Health and Care Excellence (NICE), Canadian Agency for Drugs and Technologies in Health (CADTH), Agency for Healthcare Research and Quality (AHRQ), Institute for Clinical and Economic Review (ICER), National Institute for Health and Care Research Health Technology Assessment (NIHRHTA), International Network of Agencies for Health Technology Assessment (INAHTA), Haute Autorité de Santé (HAS) and Institute for Quality and Efficiency in Health Care (IQWiG) were also screened.

The same search strategy was used to identify cost-effectiveness and HCRU studies, as current study design filters do not reliably discriminate between these types of studies [15].

### Study selection

Studies were assessed for inclusion based on the PICOTS criteria outlined in Table 1. The target population was adult patients diagnosed with RCC or UCC, according to American College of Chest Physicians (ACCP) guidelines [16]. Due to the heterogeneity in defining CC across studies, the population search strings were expanded to include all patients with CC as defined by the study investigators. Cost-effectiveness and HCRU studies were assessed against the same eligibility criteria, with the exception of the intervention and comparators. Cost-effectiveness studies were considered for inclusion if any medication known to be used for the treatment of CC, including off-label medications, compared to placebo, best supportive care, or any other intervention of interest, were reported. HCRU studies were not restricted by intervention or comparator to account for studies reporting costs and/or resource use independent of treatment effects. Outcomes of interest included costs combined with measures of effectiveness, and HCRU outcomes such as total healthcare costs, direct costs, indirect costs, out-of-pocket costs, and resource utilization. Relevant studies were limited to English language publications only, and no time or geographical restrictions were imposed.

**Table 1:** Eligibility criteria for SLR study inclusion

Criteria	Inclusion	Exclusion
Population	> 18 + years old > Have clinical evidence of CC (as defined by the study investigators) > Subgroups of interest: CC duration ≥ 1 year and < 1 year	> Patients with history of malignancy, respiratory tract infection, chronic bronchitis, or substance abuse > Currently taking an angiotensin-converting enzyme inhibitor > Immunocompromised patients > Patients with cough resulting from invasive respiratory tract instrumentation (e.g., ventilator dependent, tracheostomy, endotracheal intubation)
Interventions	Cost-effectiveness: > Gefapixant > Antitussive medications (e.g., opiates (codeine, hydrocodone), noscapine (narcotine), dextromethorphan, respiratory anesthetics (benzonatate)) > Protussive medications (e.g., expectorants (guaifenesin), mucolytic or mucus modifying agents (acetylcysteine, dornase alfa inhaled)) > Non-antitussive/non-protussive medications (e.g., antihistamines, antibiotics (azithromycin), anticholinergics, bronchodilators) > Neuromodulators/antidepressants (e.g., amitriptyline, gabapentin, baclofen, pregabalin, nortriptyline) > Inhaled corticosteroids (e.g., beclomethasone, budesonide, fluticasone, mometasone) > *Note: These treatments were eligible if given with or without a combined non-pharmacological treatment (e.g., chest physical therapy, cognitive behavioral therapy, speech therapy, behavioral cough suppression therapy, acupuncture, tai chi, yoga, meditation, aroma therapy, humidifiers, herbal tea). Additionally, studies were eligible for inclusion if patients with RCC received concomitant treatment for the underlying cause (e.g., inhaled beta2-agonists for asthma, proton pump inhibitors for gastroesophageal reflux disease)* HCRU: > Not restricted	
Comparisons	Cost-effectiveness: > Placebo or best supportive care > Any intervention of interest HCRU: > No restricted	
Outcomes	Cost-effectiveness: > Costs combined with clinical endpoints (e.g., clinical outcomes, utilities, QALYs, resource use, burden of illness) expressed in incremental costs, incremental cost-effectiveness ratios, QALYs, or any other measure of effectiveness reported together with costs HCRU: > Total healthcare costs (both direct and indirect costs) > Direct costs (e.g., costs for drugs, inpatient, outpatient, emergency room, procedures, physician visits, diagnostic/screening services, rehabilitation in a facility or at home, community-based services, medical devices, aids and appliances, alternative care) > Indirect costs (e.g., societal costs, patient productivity loss, caregiver absenteeism i.e., cost of caregiver taking time off paid work to provide care) > Out-of-pocket costs (e.g., copayments for drugs, specialty assistive devices, special transportation) > Resource utilization	
Time	> Not restricted	
Study design	Cost-effectiveness: > Full economic evaluations - Cost-effectiveness analyses - Cost utility analyses - Cost-benefit analyses - Cost consequence studies - Cost minimisation analyses > HTAs > Pooled analyses presenting cost or resource use estimates > Literature reviews summarizing results of primary research studies and/or economic evaluations HCRU: > Full economic evaluations - Cost-effectiveness analyses - Cost utility analyses - Cost-benefit analyses - Cost consequence studies - Cost minimization analyses > Partial economic evaluations - Budget impact models - Non-comparative economic studies (e.g., cost of illness studies) > Observational studies - Prospective and retrospective cohort studies - Case-control studies - Cross-sectional studies - Controlled and uncontrolled longitudinal studies - Controlled before-and-after studies - Interrupted time series studies - Historically controlled studies - Time and motion studies > Randomized controlled trials > Non-randomized clinical trials > Controlled before-and-after trials > HTAs > Pooled analyses presenting cost or resource use estimates > Literature reviews summarizing results of primary research studies and/or economic evaluations^a^	
Other	> English language only	
Region	> Global	

^a^Literature reviews involving a systematic approach to study identification and selection were of interest for the purposes of cross-referencing (e.g., SLRs, structured literature reviews, scoping reviews, landscape reviews). Narrative reviews that did not involve systematic study identification and selection or that primarily summarized an author’s viewpoints were not of interest

CC: chronic cough; HCRU: healthcare resource utilization; HTA: health technology assessment; QALY: quality-adjusted life year; RCC: refractory chronic cough; SLR: systematic literature review

Screening of all titles and abstracts identified in the search was conducted by two independent reviewers. Citations considered eligible for inclusion by both reviewers were advanced to full-text screening, which involved independent assessment of the full-text articles for inclusion by the same two reviewers. A third reviewer provided arbitration in the case of discrepancy. Each study was counted once through mapping of citations to corresponding studies.

### Data extraction and quality assessment

Data extraction from the included citations was undertaken by two independent reviewers, with a third reviewer to reach consensus for any discrepancies remaining following reconciliation. Extracted data included study identifiers, study characteristics, intervention characteristics, patient characteristics, and outcomes.

One reviewer assessed the quality of included studies, with judgments validated by a senior reviewer, using the Drummond checklist [17].

## Results

### Identification of studies

#### Cost-effectiveness

**Fig. 1:**
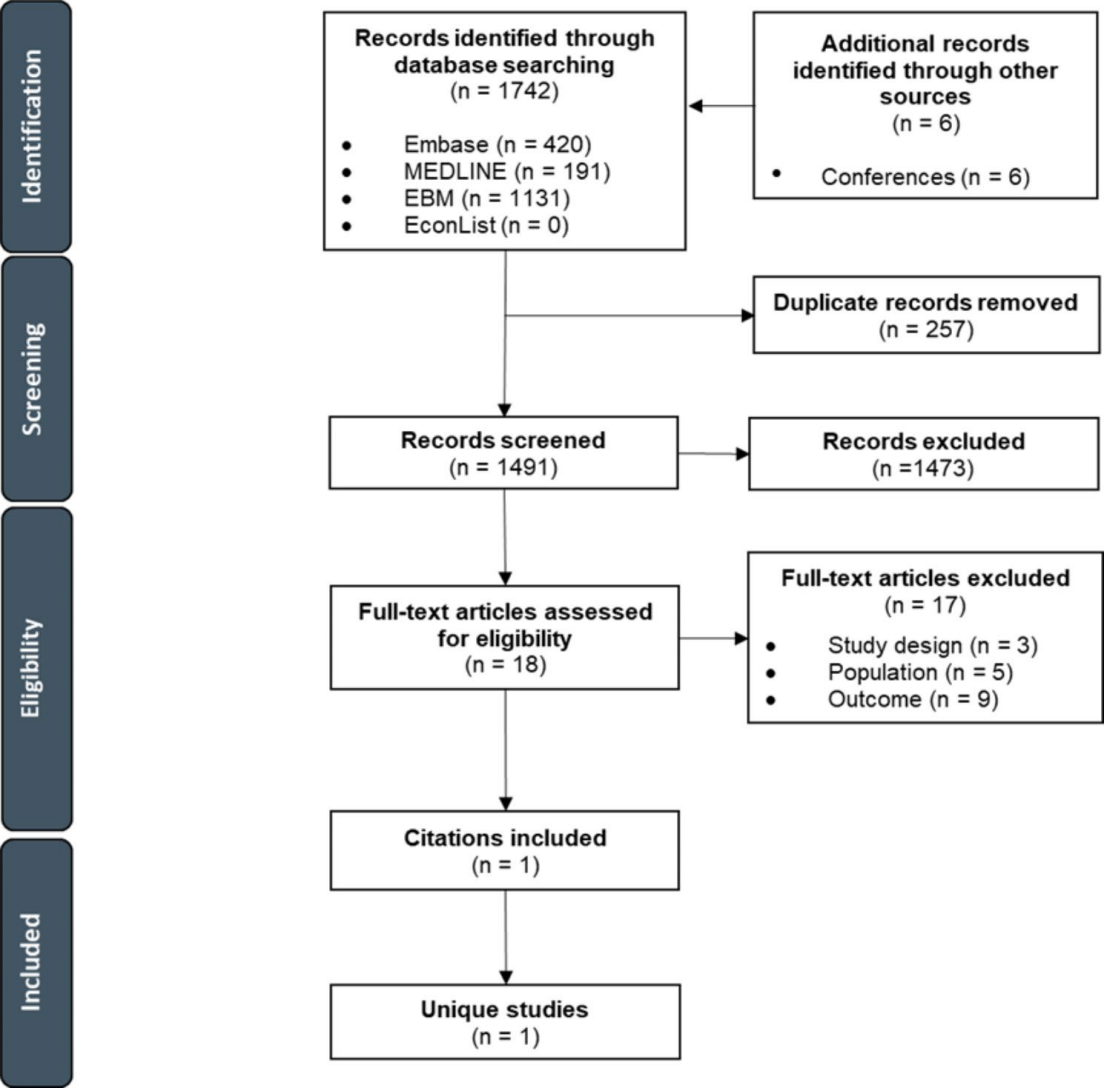
PRISMA flow diagram for cost-effectiveness studies

A total of 1742 cost-effectiveness citations were identified through electronic databases. Supplementary searches of conference proceedings and HTA websites identified a further six citations. After removal of 257 duplicates, the titles and abstracts of 1491 unique citations were screened, of which 18 were retrieved for full-text review. The majority of citations excluded at the abstract screening stage (829/1473) were excluded based on population. Only one of the 18 studies advanced to full-text review was determined to meet the inclusion criteria. Of those excluded, nine were excluded based on outcome, five based on population, and three based on study design. Due to limited evidence, data were unable to be stratified based on subgroups of interest. A PRISMA flow diagram for the study selection process is presented in Fig. 1.

#### HCRU and costs

**Fig. 2:**
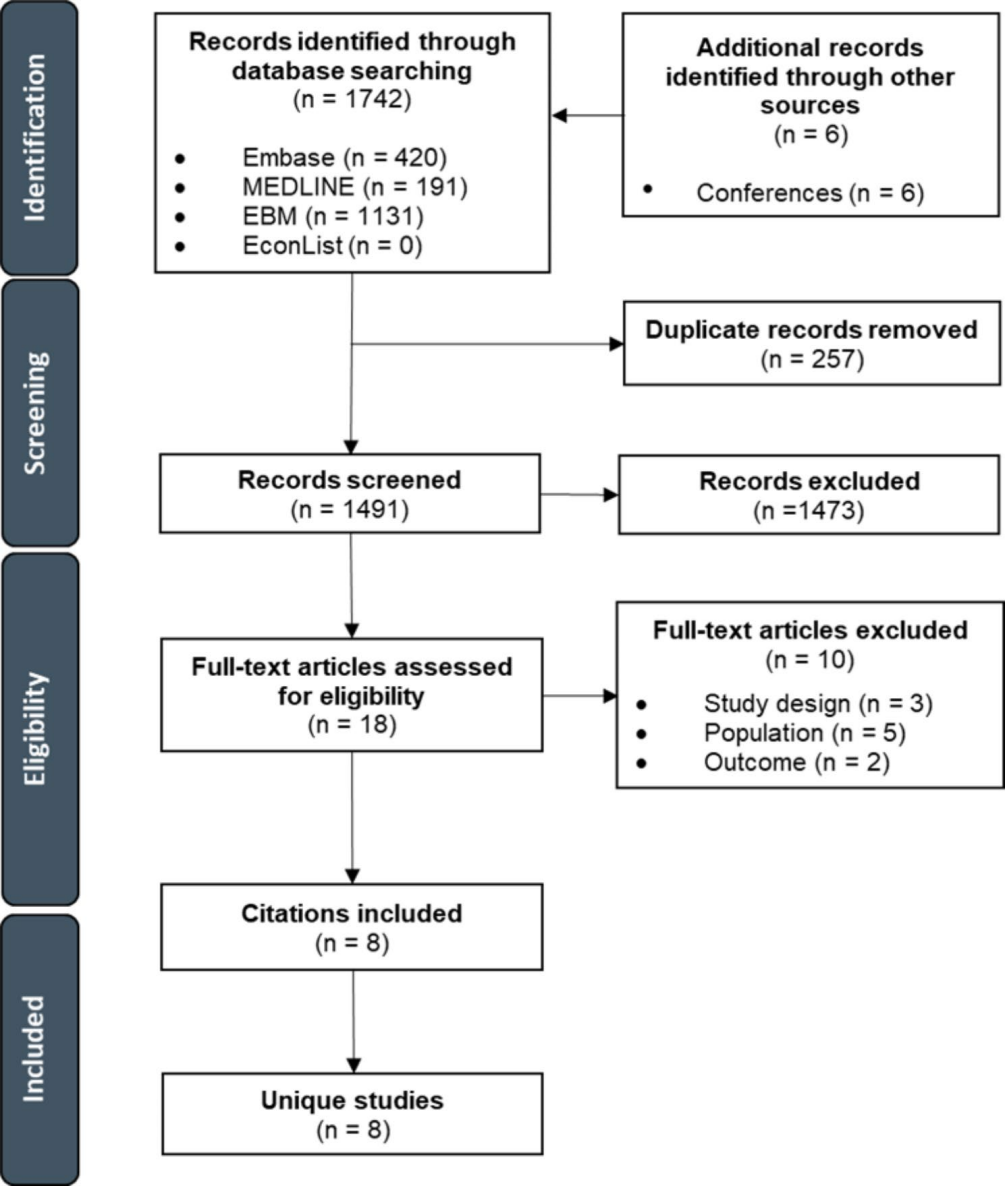
PRISMA flow diagram for healthcare resource utilization studies

Both HCRU and cost-effectiveness searches were undertaken using the same search strategy, therefore the same number of citations were identified through electronic database searches (n = 1742) and supplementary searches (n = 6) for HCRU. The titles and abstracts of 1491 unique citations were screened, of which 18 were retrieved for full-text review. The majority of citations excluded at the abstract screening stage (829/1473) were excluded based on population. Following full-text review, ten records were excluded, yielding a total of eight unique studies reporting HCRU and costs. No studies focusing on costs or HCRU in patients with a CC diagnosis of ≥ 1 year or < 1 year were identified. Of those excluded, five were excluded based on population, three based on study design, and two based on outcome. A PRISMA flow diagram for the study selection process is presented in Fig. 2.

An overview of the included studies is presented in Table 2.

**Table 2:** Identified studies reporting cost-effectiveness, HCRU and/or costs in CC

Study	Country	Study type	Intervention	Population	Sample size (n)	Time horizon	Key outcomes
Lin 2001 [18]	Singapore	CEA – decision tree	Test all then treat; Treat all; Treat sequentially Treat PNDS, test asthma, treat GERD; Treat sequentially starting with PNDS; Test then treat sequentially; Treat PNDS, test asthma and GERD Test then treat sequentially	UCC (> 3 weeks)	NR	NR	Cost-effectiveness of management strategies, duration of treatment, direct costs
Birring 2020 [19]	UK	Retrospective	NR	Persistent CC and acute cough	Persistent CC n = 12,513 Acute cough n = 137,718	2014–2015	Healthcare contacts and prescriptions, direct costs
Siu Pan Cho 2020 [11]	UK	Prospective	NR	CC	n = 100	NR	Direct costs
Meltzer 2020 [20]	US	Survey	NR	CC (> 8 weeks)	n = 296	NR	Number of diagnostic tests, evaluation in primary care, evaluation by specialist(s)
Patton 2015 [10]	US	Quality improvement	Redesign of cough clinical care process vs. usual care	CC (> 8 weeks)	n = 135	2011–2014	Specialist referrals per patient, length of patient itinerary, direct costs
Weiner 2019 [21]	US	Retrospective	NR	CC (> 8 weeks) and non-CC patients	CC n = 25,593 Non-CC n = 445,116	NR	Prescription of OCCS
Weiner 2020 [22]	US	Retrospective	NR	CC (three medical encounters with cough spanning at least 56 days within 120 days)	n = 25,767	2005–2015	Specialist referrals
Zeiger 2019 [12]	US	Retrospective	NR	CC (outpatient visit to specialist with a KPSC specific internal encounter code of CC and no dispensed ACE inhibitor)	n = 11,290	2013–2016	ED visits, hospitalizations, specialty visits, imaging, medications

ACE: angiotensin-converting enzyme; CC: chronic cough; CEA: cost-effective analysis; ED: emergency department; GERD: gastroesophageal reflux disease; KPSC: Kaiser Permanente Southern California; OCCS: opioid-containing cough suppressants; PNDS: postnasal drip syndrome; NR: not reported; UCC: unexplained chronic cough; UK: United Kingdom; US: United States

### Cost-effectiveness analyses

The single cost-effectiveness analysis identified for CC was conducted in Singapore and published as a journal article in 2001 [18]. The study involved a decision tree model to assess the cost-effectiveness of six alternative management strategies for UCC lasting at least three weeks: [1] Test all then treat; [2] Treat all; [3] Treat postnasal drip syndrome (PNDS), test asthma, treat gastroesophageal reflux disease (GERD); [4] Treat sequentially starting with PNDS; [5] Test then treat sequentially; and [6] Treat PNDS, test asthma and GERD together. Treat all, Test then treat sequentially, and Treat sequentially starting with PNDS were found to be the most cost-effective strategies considering the expected duration of cough versus expected cost, with direct costs of $157, $149, and $184 USD, respectively [18].

### Costs associated with CC

There is a paucity of cost data in CC, with only four studies reporting costs, as outlined in Table 3. Of these, two were conducted in the United Kingdom (UK), [11, 19] one in the United States (US), [10] and one in Singapore [18]. Two studies were published as journal articles, whilst two were presented as conference abstracts. Of note, a retrospective study comparing CC and acute cough in the UK found that patients with CC incurred higher costs than those with acute cough (all healthcare contact, per person-year equaled £3,663 in patients with CC and £2,700 in acute cough) [19]. The impact of redesigning the cough clinical care process in the US was assessed and it was proposed that by introducing a multidisciplinary, collaborative approach to care, the costs could be nearly halved ($656 vs. £$1,319 USD in current usual care) [10]. No studies explored indirect costs associated with CC.

**Table 3:** Summary of studies reporting costs associated with CC

Study	Country	Study type	Population	Sample size (n)	Intervention	Direct cost	Cost year
Lin 2001 [18]	Singapore	CEA – decision tree	UCC (> 3 weeks)	NR	Treat all	$157 USD	NR
					Test then treat sequentially	$149 USD	NR
					Treat PNDS, test asthma, treat GERD	$184 USD	NR
					Treat PNDS, test asthma and GERD	$280 USD	NR
					Test then treat sequentially	$516 USD	NR
					Test all then treat	$556 USD	NR
Birring 2020 [19]	UK	Retrospective	CC and acute cough	CC n = 12,513 Acute cough n = 137,718	NR	CC patients incurred more costs than acute cough patients, with the greatest disparity in primary care	2014–2015
Siu Pan Cho 2020 [11]	UK	Prospective	CC	n = 100	NR	£1,800 GBP	NR
Patton 2015 [10]	US	Quality improvement	CC (> 8 weeks)	n = 135	Redesign of cough clinical care process	$656 (SD: $297) USD	2011–2013
					Usual care	$1,319 (SD: $341) USD	2011–2013

CC: chronic cough; CEA: cost-effectiveness analysis; GBP: British pound; GERD: gastroesophageal reflux disease; NR: not reported; PNDS: postnasal drip syndrome; SD: standard deviation; UCC: unexplained chronic cough; UK: United Kingdom; US: United States; USD: United States dollar

### HCRU associated with CC

Resource use was explored in seven studies, as outlined in Table 4. HCRU in the US was evaluated in five studies, [10, 12, 20–22] whilst one study assessed HCRU in the UK, [19] and one in Singapore [18]. Only two studies were published as journal articles, whilst the remaining five were conference abstracts. Types of resource use reported included healthcare contacts and prescriptions, diagnostic tests, referrals and specialist evaluations, and treatment use. Differences in the resource use reported across studies prevent cross-country comparisons, however it is clear that resource use is high in patients with CC. A retrospective study in the US compared patients with and without CC and found an increase in prescription of opioid-containing cough suppressants (OCCS) in patients with CC (60 prescriptions per 100-patients in CC cohort vs. 12 prescription per 100-patients in non-CC cohort) [21]. 35% of patients with CC in this study reported ≥ 3 OCCS prescriptions, with 0.6% reporting ≥ 10 OCCS prescriptions [21]. Data collected from a retrospective database study identified chest x-rays (80.3%), systemic respiratory antibiotics (72.4%) and narcotics, including codeine (60.9%) as the most common causes for healthcare interactions [12].

**Table 4:** Summary of studies reporting HCRU associated with CC

Study	Country	Study type	Population	Sample size (n)	Intervention	Resource description	Resource use
Lin 2001 [18]	Singapore	CEA – decision tree	UCC (> 3 weeks)	NR	Treat all	Duration of treatment	5 weeks
					Treat sequentially		6 weeks
					Treat PNDS, test asthma, treat GERD		6 weeks
					Treat PNDS, test asthma and GERD		6 weeks
					Test then treat sequentially		5 weeks
					Test all then treat		4 weeks
Birring 2020 [19]	UK	Retrospective	CC and acute cough	CC n = 12,513 Acute cough n = 137,718	NR	Healthcare contacts and prescriptions, direct costs	CC pts used more resources than acute cough pts, with the greatest disparity in primary care
Meltzer 2020 [20]	US	Survey	CC (> 8 weeks)	n = 296	NR	Number of diagnostic tests (mean)	2.1
						Evaluation in primary care	90% of patients
						Evaluation by specialist(s)	52% of patients
Patton 2015 [10]	US	Quality improvement	CC (> 8 weeks)	n = 135	Redesign of cough clinical care process vs. usual care	Referrals per patient (mean)	Intervention: 1.2 Comparator: 3.3
						Length of patient itinerary	Intervention: 12.9 days Comparator: 126.9 days
Weiner 2019 [21]	US	Retrospective	CC (> 8 weeks) and non-CC patients	CC n = 25,593 Non-CC n = 445,116	NR	Prescription of OCCS 1 year after index date	22% of CC pts, 6% of non-CC pts
						Number of OCCS prescriptions per 100 patients	CC: 60 Non-CC: 12
						Prescription of ≥ 3 OCCS	35% of CC pts out of all CC prescribed OCCS
						Prescription of ≥ 10 OCCS	0.6% of CC pts out of all CC prescribed OCCS
						Payer of OCCS for CC patients	Medicaid: 39%, Medicare: 19%, Commercial insurance: 16%
Weiner 2020 [22]	US	Retrospective	CC (three medical encounters with cough spanning at least 56 days within 120 days)	n = 25,767	NR	Specialist referrals	3.1%
						Delay from index date to first referral (mean)	63 days
						First referral type	Pulmonary: 1.9% Otolaryngology: 0.9% Allergy: 0.2% Gastroenterology: 0.1%
						Multiple referrals	Two specialties: 0.2% Three specialties: <0.1% Four specialties: <0.1%
Zeiger 2019 [12]	US	Retrospective	CC (outpatient visit to specialist with a KPSC specific internal encounter code of CC and no dispensed ACE inhibitor)	n = 11,290	NR	ED visit	28.5% of patients
						Hospitalization	9.8% of patients
						Two or more different specialty visits	46.7% of patients
						Chest x-ray	80.3% of patients
						Advanced chest imaging	21.2% of patients
						Narcotics, including codeine	60.9% of patients
						Antitussives, including codeine	58.9% of patients
						Oral corticosteroids	46.8% of patients
						Systemic respiratory antibiotics	72.4% of patients
						PPIs	45.0% of patients
						Antidepressants	26.0% of patients
						Neuromodulators	13.9% of patients

ACE: angiotensin-converting enzyme; CC: chronic cough; CEA: cost-effective analysis; ED: emergency department; GERD: gastroesophageal reflux disease; OCCS: opioid-containing cough suppressants; PNDS: postnasal drip syndrome; PPI: proton pump inhibitor; NR: not reported; UCC: unexplained chronic cough; UK: United Kingdom; US: United States

### Study quality assessment

The quality of the eight studies included in the two SLRs was assessed using the Drummond checklist in terms of their clarity of reporting in ten different areas: study question, selection of alternatives, form of evaluation, effectiveness data, benefit measurement, costing, modelling, analysis and interpretation of results, allowance for uncertainty, presentation of results [17]. Overall, the quality of the included studies was moderate to low, often because the short length of conference abstracts did not allow for complete reporting.

## Conclusions

There is a paucity of literature on HCRU and costs in CC, and very limited cost-effectiveness analyses in this population possibly due to lack of approved therapies for CC. The findings of this SLR indicate that the economic burden appears to be higher in patients with CC however, without direct comparison to the general population is it difficult to conclude on the level of impact. The increased burden may be expected due to challenges with diagnosis which may result in multiple physician visits and referrals, an increased number of comorbidities in patients with CC compared to patients without CC, and lack of approved treatments. It is likely that any current estimates of the burden in patients is underestimated due to widespread underdiagnosis. However, limited conclusions can be drawn in the absence of further data. Future studies involving cough registries might help to better quantify the HCRU and costs attributable to patients with CC.

## Discussion

CC is a highly burdensome condition affecting approximately 10% of the adult population [5]. Symptoms of CC include chest pains, sleep disturbance and hoarse voice, and in severe cases, syncope, stress incontinence and vomiting [3]. Despite the high burden CC places on patients, diagnosis of CC remains a challenge, causing significant delays in treatment [6, 16]. As a result, CC is expected to have a high economic burden including both direct costs such as costs for physician visits, medication, diagnosis and hospitalization, and indirect costs including loss of productivity and absenteeism from work. This SLR was performed to identify and describe current literature on the economic burden of RCC and UCC, including the relative cost-effectiveness of current treatments.

The SLR revealed that there is a lack of evidence reporting the economic burden of CC, with notable gaps in the reporting of out-of-pocket expenses, costs for over-the-counter medication, and the costs associated with discrete treatments. Overall, only six conference abstracts and two journal articles were identified reporting on the economic impact of CC. Only one study focused on UCC, [18] and no studies evaluated RCC. Although one cost-effectiveness analysis was identified, no studies reported incremental costs, incremental life years, or incremental QALYs [18]. Notably, the majority of citations were excluded based on population, further emphasizing the lack of evidence in this disease area, and the heterogeneity in defining CC. Whilst the limited data on the economic impact of CC may reflect difficulties in collecting and analyzing such data, for example due to diagnostic challenges, there is a clear need for future studies to focus on quantifying this burden. Following the completion of this SLR, the ICD-10 Coordination and Maintenance Committee in the US implemented a new ICD-10-CM code in October 2021 specific for the diagnosis of CC (R05.3) [13] This addition may address some of the difficulty in identifying the economic burden of patients with CC however, until there is consistent implementation and diagnoses with a standardized code there will remain an uncertainty in the true economic burden of CC.

Despite the limited evidence base, collectively it is shown that patients with CC use more healthcare resources and incur greater costs than patients with acute or non-CC [19]. Although most patients with CC are managed in a primary care setting, up to half of patients are referred to one or more specialists [12, 20, 22]. When compared to patients without CC, a recent population-based study confirmed that patients with CC encountered more (1.5 times) visits to health care providers compared to a non-CC control group (6.7 vs. 4.4; p = < 0.001) [9]. Patients with CC receive an average of 2.1 diagnostic tests, with most undergoing chest x-ray and many undergoing advanced chest imaging [20]. As many as half of patients with CC are treated with opiates or other narcotics, which is of pressing concern given that overreliance on opioid prescriptions is a driving factor of the current opioid crisis [12, 21, 23]. From a financial perspective, the average direct cost incurred by patients with CC was reported to be £1,800 GBP over a 12 month period in the UK and $1,319 in the US [10, 11]. However, these data are based on individual studies and so should be taken with caution. Calculating cost-effectiveness in CC using traditional measures (i.e., number of hospitalizations) poses its own difficulty as patients with CC are typically not hospitalized due to CC itself, this is often attributed to a comorbid condition. No studies reported on the indirect costs incurred. Further, the broad definition of CC and heterogeneity among patients, compounded by the high prevalence of comorbidities, causes challenges in estimating costs directly attributable to CC. It is difficult to compare these costs to the general population as no studies examined the costs for patients with CC in the context of the general population.

### Strengths and limitations

The SLR involved highly sensitive database searches of peer-reviewed literature as well as searches of recent conferences and HTA body websites to identify unpublished studies. Validated search filters recommended by SIGN and InterTASC were used to prioritize sensitivity over specificity of the searches [14, 15]. However, as with any SLR, there is a risk that studies published after the database searches conducted in February 2021 may not have been captured.

A particular weakness of this SLR is that the limited amount of available published data prevents comprehensive or definitive conclusions from being drawn on the economic impact of CC. The variation in follow-up duration, ranging from weeks to years, prevents an accurate characterization of costs and healthcare resource utilization. The bulk of the evidence included in this SLR came from conference abstracts, which do not provide complete information and should be interpreted with caution as they do not undergo the same methodologically rigorous peer review process as fully published results. Further, CC involves long-term management, and there is a clear lack of studies to adequately assess the true long-term costs of the condition.

Of note, there are difficulties in identifying relevant data and performing appropriate analysis, given the broad definition of CC and challenges with diagnosis. In this SLR, the target population was adult patients diagnosed with CC, either refractory or unexplained, according to ACCP guidelines [16]. However, broader inclusion criteria were applied to ensure capture of all relevant data. Studies evaluating any type of CC as defined by the study investigators regardless of its duration, including idiopathic CC, as well as studies using alternative definitions of RCC or UCC, were also included. Further, it can be difficult to isolate the burden specifically associated with CC due to the high prevalence of comorbidities in these patients. In previous studies it was noted patients with CC were more likely to be smokers and/or obese, and to have respiratory or airway diseases, psychological disorders, diabetes, or chronic pain [24]. These comorbidities are likely to attribute to greater medical attention and costs.

Overall, whilst evidence is limited, CC is associated with high utilization of healthcare resources involving multiple patient referrals, diagnostic tests, and drug prescriptions. Combined with the delayed diagnosis and limited treatment options in this population, there is high humanistic and economic burden, with a remaining unmet need for a more effective treatment approach to reduce the associated burden.

### Take home message

Patients with CC encounter multiple physicians and specialists to seek a definitive diagnosis, resulting in delayed diagnosis and appropriate treatment. This SLR reported a paucity of economic data; however, it found patients with CC to incur higher costs and more resource use than those with acute cough.

### Electronic supplementary material

Below is the link to the electronic supplementary material.

Supplementary Material 1

## Data Availability

All data generated or analyzed during this study are included in this published article and its supplementary information files.
