# Supplementary material for: Understanding the economic burden of chronic cough: a systematic literature review
Source: BMC Pulm Med. 2023 Oct 31;23:416. doi: 10.1186/s12890-023-02709-9 (PMC10619292; doi:10.1186/s12890-023-02709-9)
Supplement: Supplementary file 1 — Supplementary Material 1 [file 12890_2023_2709_MOESM1_ESM.docx]

Supplementary Table 1. EMBASE search strategy

| No. | Terms |
| --- | --- |
| 1 | exp chronic cough/ |
| 2 | ((chronic adj3 cough) or (unexplained adj3 cough) or (idiopathic adj3 cough) or (refractory adj3 cough) or (unexplained adj3 cough) or (intractable adj3 cough) or (persistent adj3 cough) or (cough adj3 syndrome)).mp. |
| 3 | 1 or 2 |
| 4 | Socioeconomics/ |
| 5 | Cost benefit analysis/ |
| 6 | Cost effectiveness analysis/ |
| 7 | Cost of illness/ |
| 8 | Cost control/ |
| 9 | Economic aspect/ |
| 10 | Financial management/ |
| 11 | Health care cost/ |
| 12 | Health care financing/ |
| 13 | Health economics/ |
| 14 | Hospital cost/ |
| 15 | (fiscal or financial or finance or funding).tw. |
| 16 | Cost minimization analysis/ |
| 17 | (cost adj estimate$).mp. |
| 18 | (cost adj variable$).mp. |
| 19 | (unit adj cost$).mp. |
| 20 | or/4-19 |
| 21 | (econom$ or cost or costs or costly or costing or price or prices or pricing or pharmacoeconomic$).ti,ab. |
| 22 | or/20-21 |
| 23 | 3 and 22 |
| 24 | limit 24 to English language |

Supplementary Table 2. MEDLINE search strategy

| No. | Terms |
| --- | --- |
| 1 | Economics/ |
| 2 | costs and cost analysis/ |
| 3 | Cost allocation/ |
| 4 | Cost-benefit analysis/ |
| 5 | Cost control/ |
| 6 | Cost savings/ |
| 7 | Cost of illness/ |
| 8 | Cost sharing/ |
| 9 | deductibles and coinsurance/ |
| 10 | Medical savings accounts/ |
| 11 | Health care costs/ |
| 12 | Direct service costs/ |
| 13 | Drug costs/ |
| 14 | Employer health costs/ |
| 15 | Hospital costs/ |
| 16 | Health expenditures/ |
| 17 | Capital expenditures/ |
| 18 | Value of life/ |
| 19 | exp economics, hospital/ |
| 20 | exp economics, medical/ |
| 21 | Economics, nursing/ |
| 22 | Economics, pharmaceutical/ |
| 23 | exp "fees and charges"/ |
| 24 | exp budgets/ |
| 25 | (low adj cost).mp. |
| 26 | (high adj cost).mp. |
| 27 | (health?care adj cost$).mp. |
| 28 | (fiscal or funding or financial or finance).tw. |
| 29 | (cost adj estimate$).mp. |
| 30 | (cost adj variable).mp. |
| 31 | (unit adj cost$).mp. |
| 32 | (economic$ or pharmacoeconomic$ or price$ or pricing).tw. |
| 33 | or/1-32 |
| 34 | (economic$ or cost or costs or costly or costing or price or prices or pricing or pharmacoeconomic$).ti,ab. |
| 35 | (expenditure$ not energy).ti,ab. |
| 36 | budget$.ti,ab. |
| 37 | (economic* or cost or costs or costly or costing or price or prices or pricing or pharmacoeconomic* or pharmaco-economic* or expenditure or expenditures or expense or expenses or financial or finance or finances or financed).ti,kf. |
| 38 | 33 or (or/34-37) |
| 39 | chronic cough.mp. |
| 40 | ((chronic adj3 cough) or (unexplained adj3 cough) or (idiopathic adj3 cough) or (refractory adj3 cough) or (unexplained adj3 cough) or (intractable adj3 cough) or (persistent adj3 cough) or (cough adj3 syndrome)).mp. |
| 41 | 39 or 40 |
| 42 | 38 and 41 |
| 43 | limit 42 to English language |

Supplementary Table 3. EconLit search strategy

| No. | Terms |
| --- | --- |
| 1 | chronic cough.mp. |
| 2 | ((chronic adj3 cough) or (unexplained adj3 cough) or (idiopathic adj3 cough) or (refractory adj3 cough) or (unexplained adj3 cough) or (intractable adj3 cough) or (persistent adj3 cough) or (cough adj3 syndrome)).mp. |
| 3 | or/1-2 |
| 4 | limit 3 to English language |

Supplementary Table 4. EBM Reviews search strategy

| No. | Terms |
| --- | --- |
| 1 | chronic cough.mp. |
| 2 | ((chronic adj3 cough) or (unexplained adj3 cough) or (idiopathic adj3 cough) or (refractory adj3 cough) or (unexplained adj3 cough) or (intractable adj3 cough) or (persistent adj3 cough) or (cough adj3 syndrome)).mp. |
| 3 | or/1-2 |
